# Climate-Resilient Dairy Cattle Production: Applications of Genomic Tools and Statistical Models

**DOI:** 10.3389/fvets.2021.625189

**Published:** 2021-04-29

**Authors:** Mullakkalparambil Velayudhan Silpa, Sven König, Veerasamy Sejian, Pradeep Kumar Malik, Mini Ravi Reshma Nair, Vinicius F. C. Fonseca, Alex Sandro Campos Maia, Raghavendra Bhatta

**Affiliations:** ^1^Institute of Animal Breeding and Genetics, Justus-Liebig-Universität Gießen, Gießen, Germany; ^2^Center for Climate Resilient Animal Adaptation Studies, Indian Council of Agricultural Research-National Institute of Animal Nutrition and Physiology, Bangalore, India; ^3^Innovation Group of Thermal Comfort and Animal Welfare (INOBIO-MANERA), Animal Science Department, Universidade Federal da Paraíba, Areia, Brazil; ^4^Brain Function Research Group, Faculty of Health Sciences, School of Physiology, University of the Witwatersrand, Johannesburg, South Africa; ^5^Innovation Group of Thermal Comfort and Animal Welfare (INOBIO-MANERA), Faculdade de Ciências Agrárias e Veterinárias, Universidade Estadual Paulista (Unesp), São Paulo, Brazil

**Keywords:** dairy (cows), heat stress, molecular markers, thermoregulation, genome-wide association study

## Abstract

The current changing climate trend poses a threat to the productive efficacy and welfare of livestock across the globe. This review is an attempt to synthesize information pertaining to the applications of various genomic tools and statistical models that are available to identify climate-resilient dairy cows. The different functional and economical traits which govern milk production play a significant role in determining the cost of milk production. Thus, identification of these traits may revolutionize the breeding programs to develop climate-resilient dairy cattle. Moreover, the genotype–environment interaction also influences the performance of dairy cattle especially during a challenging situation. The recent advancement in molecular biology has led to the development of a few biotechnological tools and statistical models like next-generation sequencing (NGS), microarray technology, whole transcriptome analysis, and genome-wide association studies (GWAS) which can be used to quantify the molecular mechanisms which govern the climate resilience capacity of dairy cows. Among these, the most preferred option for researchers around the globe was GWAS as this approach jointly takes into account all the genotype, phenotype, and pedigree information of farm animals. Furthermore, selection signatures can also help to demarcate functionally important regions in the genome which can be used to detect potential loci and candidate genes that have undergone positive selection in complex milk production traits of dairy cattle. These identified biomarkers can be incorporated in the existing breeding policies using genomic selection to develop climate-resilient dairy cattle.

## Introduction

With the growing global human population, a huge demand for livestock products can be expected in the coming years (1). At the same time, the global climatic conditions are predicted to become warmer and diverge across the continents (2). According to the climate change models, the mean global temperature may be 2.6–4.8°C warmer by 2100 as compared to the conditions that prevailed in 2010 (2). This situation could thus lead to heat stress, which is a potent factor negatively influencing livestock production as it alters water availability, the quality of forage and roughage, and the production and reproduction characteristics and health status of the animals (3). Among all the domesticated production animals, dairy cows are most susceptible to heat stress as a result of the intensive long-term breeding done in them so as to improve their milk production, which has led to a higher metabolic heat generation in these animals (4). Furthermore, in tropical countries, where the impact of climate change is predicted to be more severe, dairy cattle are primarily reared in an extensive system, unlike that of chicken and pig which follow a semi-intensive system of rearing. This further contributes to the greater susceptibility of dairy cows to heat stress. In order to dissipate excess heat during thermal stress, the animal exhibits various physiological and metabolic adaptive mechanisms which are energy consuming, and this is believed to cause a proportional decline in milk yield in these animals (5). Furthermore, heat stress was also reported to alter the milk composition through reduction in total protein content and total fat content in milk (6).

Milk production is one of the most important economic traits in cattle which, being polygenic, is affected by many genes. Advancements in the field of molecular genetics have aided in identifying various candidate genes and quantitative trait loci (QTL) regions which were found to have an association with milk yield, fat yield, protein yield, and other milk production traits (7). This information on genomic sequence variation has been applied widely in livestock improvement schemes as they are backed up with a supporting proof of genotypic data and its confirmed association (8, 9).

Apart from the genetic factors, environmental factors also influence milk production, among which ambient temperature is one of the most important abiotic factors (10). An annual milk loss of ~2% of the total milk production of the country was reported due to thermal stress on the animal (10). It is also estimated that the loss in milk production due to global warming would rise up to 3.2 million tons by 2020 and would exceed 15 million tons by 2050 (10). This statistic is quite alarming, and this warrants developing suitable mitigation strategies to reverse the condition. The ability of the cattle to perform normal biological functions in various adverse environmental conditions denotes its resilient capacity (11). Although several management and nutritional strategies are available to ameliorate the heat stress impact on livestock, still these strategies may not be offering a permanent solution to the issue. Hence, a better understanding of the genetic differences and molecular mechanisms involved in thermo-tolerance and innate resilience is necessary. In a couple of recent review articles on the subject, emphasis was given to assess climate resilience generally in ruminant species, and negligible reports are available on the subject with respect to establishing climate resilience in dairy cattle (3, 11–13). Furthermore, not many reviews were attempted, in particular on the application of genomic tools and statistical models, to establish climate resilience in dairy cattle. Hence, generating and synthesizing information from various research reports (14–16) in this line could be very useful and may provide vital information for improving climate resilience in dairy cattle. Such information could be very useful for designing future breeding strategies to establish climate resilience in dairy cattle.

With this background, this review was targeted to synthesize information pertaining to the application of various advanced genomic tools and statistical models that are currently available to identify climate-resilient dairy cows.

## World Milk Production Statistics

Globally, around 15 million households are engaged in milk production, as it provides food security and nutrition and is also an important source of income especially for small-scale producers (17). More than 61% increase in world milk production was observed over the last three decades from 522 million tones (MT) in 1987 to 843 MT in 2018 (17). It is the developing countries that have contributed the most for this increase, and among them is India, being the world's largest milk producer that have contributed to 21% of the global milk production (18).

## Factors Influencing Cow Milk Production

Milk production in dairy cattle are predominantly governed by genetic and environmental factors, and thus this particular trait has been reported to be influenced by various factors such as breed (19), genetic make-up (7), parity or lactation order (19), age, dietary composition, season (20), heat stress (6, 20), and differences in agro-ecological zones. Thus, it is of utmost importance to consider these factors while selecting the suitable breed(s) to improve milk production.

An important genetic variable, giving a better understanding of the variation in a trait and the possibility of genetic improvement, is heritability (*h*^2^). In general, milk production traits have low to medium heritability ranging from 0.17 to 0.30 (21). Suzuki and Van Vleck (21) reported an average heritability estimate for milk, fat, protein, and SNF yields as 0.30 ± 0.1, 0.30 ± 0.1, 0.26 ± 0.1, and 0.27 ± 0.1, respectively, in Japanese Holstein cows. Additionally, the heritability for milk yield was also found to be influenced by environmental factors depicting a genotype–environment interaction. Lee et al. (22) reported a mild decline in *h*^2^ for milk yield of Holstein cattle under heat stress in South Korea. Furthermore, they observed an increased *h*^2^ at a temperature–humidity index (THI) of 79. In general, the *h*^2^ estimates for milk yield in these cattle ranged from 0.111 to 0.176. Similarly, Kimiya et al. (23) estimated the genetic parameters of the milk production trait of Iranian cattle under heat stress. As per their report, the *h*^2^ of milk production ranged from 0.1 to 0.22. Additionally, THI and milk production was found to be positively correlated within a range of 0.1–0.9.

Feeding management in cattle not only minimizes nutrient wastage but also maximizes milk production. Sherasia et al. (24) observed that feeding nutritionally balanced diet to cattle improved their milk yield, 4% fat-corrected milk yield, and milk protein percentage by 3.6, 5.0, and 3.3%, respectively. Thus, this factor is also of utmost importance for improving milk production in cattle.

Among the environmental factors affecting milk production, heat stress is of a major concern (25). So as to maintain homeostasis, the animal has to make several physiological adjustments which bring about changes in its feeding pattern, rumen functions, and also udder health, thereby resulting in a reduction in its milk production efficiency (26).

## Heat Stress Impact on Milk Yield and Composition

Heat stress has direct and indirect impact on dairy cattle, affecting both milk production and milk quality. Increasing temperature and/or humidity leads to reduced feed intake in animals, thereby causing a reduction in most of the production activities (25). During dry period, heat stress also affects the mammary gland proliferation and development of the gland, ultimately leading to a decrease in milk yield (27). In dairy cattle, this situation leads to a significant drop in milk production especially in high producers. The decline in milk production due to heat stress could be to the tune of nearly 10–15% reduction in farms following cooling practices, and the reduction can go up to 40–50% when no cooling management was practiced. Subjecting 12 lactating dairy goats to heat stress in a climatic chamber reveal 53% decline in milk yield on day 4 of the exposure (28). Heat stress affects not only milk quantity but also milk quality (29). Bernabucci et al. (30) studied the effect of hot season on milk protein fraction and observed a lower milk yield (−10%), casein percentage (2.18 vs. 2.58%), and casein number (72.4 vs. 77.7%) in summer as compared to spring. Heat stress also alters the milk lipid profile, wherein significant changes in the triacylglycerol and polar lipid profiles were reported by Liu et al. (4). Thus, protecting the productive performance of cattle from the adverse effect of climate change is a rising concern, and suitable measures have to be taken in developing thermal-resilient cattle breeds.

## Heat Stress Associated With Economic Loss in the Dairy Industry

The global livestock sector is highly dynamic and is a significant asset holding a value of at least 1.4 trillion USD (31). Its share in agricultural gross domestic products has already touched 33% and is growing fast (31). The livestock system, however, does get negatively affected by the adversities of changing climatic conditions. Various studies have reported a negative correlation between heat stress and milk production characteristics (32, 33). This decrease in production has led to significant economic losses, too. St-Pierre et al. (32) reported an annual economic loss ranging between 1.69 and 2.36 billion USD in the United States due to the impact of heat stress on the dairy industry. Moreover, it was also predicted that, with the rising global warming scenario, heat stress would further aggravate milk production losses across dairy farms in the U S, with an average decline of 0.6% in 2010 to 1.4% in 2030, which may also touch 2% in a few states (34). Furthermore, in a study conducted at University of Manitoba, Canada, heat stress was reported to reduce milk production, resulting in an economic loss of over $0.45/cow/day (35). Heat stress was also predicted to take a toll in the European countries with a projected decline in milk production by 3.5%, reflecting a monthly financial loss to the farmers of about 6.6% to the present status (36). Additionally, the third largest milk producer in the world, China, has also been projected to incur losses in milk production, which was predicted to go up to 47% by 2050 (37). Heat stress was also found to negatively influence milk production in India, accounting for an annual milk loss of 1.8 MT, which leads to an estimated economic loss of 0.38 billion USD (33). This necessitates the urgent need for finding a solution to heat-associated reduction in milk through the development of strategies for ameliorating the condition.

## Genotype × Environmental Interaction and Evolutionary Changes on Dairy Cattle Population in Hot Conditions: From Quantitative Genetic to Genomic Advances

There are reports which established uncertainty in the performance of genetically superior animals when subjected to a new environment (38, 39). Genotype × environment interaction (G × E) studies help to evaluate the level of this uncertainty and can predict the extent to which an animal may perform in the new environment (38). This interaction arises when different environments unevenly influence the different genotypes (40). Therefore, it is necessary to understand the production environment before making a selection policy since the interactions between genetic and environment may hamper the performance efficiency of the animal (38, 40). High-yielding dairy cows derived from temperate regions, for example, have failed to express their productive potential when imported to be raised under tropical conditions (41). Nevertheless, over the last centuries, the populations of dairy cattle in tropical regions have acquired phenotypic characteristics that conferred them better climate resilience and productive performance. The genetic structure of the purebred Holstein population, for example, was substantially modified over the 80 years of evolution by having phenotypic traits much different to those contemporary Holstein breed from temperate climates, especially those related to the cutaneous surface characteristics (42–44). The involvement of G × E in the population would infer a change in performance of the animals and thereby account for re-ranking in different environments (45). Therefore, it is of utmost importance to evaluate the extent of G × E for heat tolerance before implementing selection for thermo-tolerance.

There are a number of statistical models to evaluate G × E, but the two most frequent models are reaction norm (RM) model (45) and multiple trait model (46). The RM models have been used widely to estimate genetic components of heat tolerance in dairy cattle across various countries (45). This model incorporates genotype-specific random regression on environmental variables wherein the phenotype (e.g., heat tolerance trait) is expressed as a function of environmental descriptors (e.g., THI). The RM models permit to include the environmental changes on a continuous scale (47). The multiple trait model, however, considers environment to be specific to a group of herds (46).

In the study conducted by Cheruiyot et al. (45), the G × E for heat tolerance was evaluated for milk production traits in Australian Holsteins using the test-day milk yield records of cows and sires that had their progeny in different climatic regions. In their study, test-day milk yield records of cows and sires (that had progenies) from different climatic regions were selected, and the THI was used to estimate the environmental gradient prevailing in the region. The univariate RM model was used to analyze the data which were then compared with that of the multi-trait model. Several genetic variables were estimated, which revealed the heritability for milk, protein, and fat for the THI trajectory, 60 ≤ THI ≥ 75. The estimated heritability for these variables ranged from 0.13 ± 0.01 to 0.14 ± 0.009, 0.10 ± 0.007 to 0.11 ± 0.01, and 0.07 ± 0.007 to 0.09 ± 0.006, respectively. In contrast to these values, the heritability estimated for MT were greater than that from RM wherein heritability ranged from 0.21 ± 0.01, 0.14 ± 0.006, and 0.16 ± 0.01 and 0.11 ± 0.01 and 0.12 ± 0.006. The heritability estimates for milk, protein, and fat yield over THI trajectory (i.e., 60 ≤ THI ≥ 75) ranged from 0.13 ± 0.01 to 0.14 ± 0.009, 0.10 ± 0.007 to 0.11 ± 0.01, and 0.07 ± 0.007 to 0.09 ± 0.006, respectively. In contrast, the heritability estimates from MT were greater than those from RM, with values at 5th (THI = 61) and 95th (THI = 73) percentiles of 0.18 ± 0.007 and 0.21 ± 0.01, 0.14 ± 0.006 and 0.16 ± 0.01, and 0.11 ± 0.01 and 0.12 ± 0.006 for milk, protein, and fat yields, respectively. The slope of the reaction norms which estimates the estimated breeding value (EBV) for sires (having at least 100 daughters with records) ranged from −11.69 to 5.84, −3.19 to 1.96, and −2.92 to 2.29 for milk, protein, and fat yields, respectively, with the standard deviation of 2.08, 0.82, and 0.75, respectively. Of these, 65, 57, and 64% sires showed resilience for heat stress, which was reflected as consistent slopes of EBV. The contradicting trends for heritability estimates were discussed to be a possibility of different methods adopted in the two models. While the average of heterogeneous variances over the days-in-milk (DIM) was used to estimate the heritability in RM, a separate residual variance for the three traits at the 5th, 50th, and 75th percentile was used in MT. The authors also mentioned an alternative to tackle this issue, which was to incorporate (co)variance component models which would consider the interactions between DIM and THI. Such models would therefore aid to estimate residual variances and heritabilities within DIM × THI combinations.

A less dense coat with thick, short, packed, and well-settled hairs represents a low resistance to the mass and heat transfer to the surrounding environment, by which this set of combination characterizes the most tropical cattle breeds (43). Maia et al. (43, 48) studied the coat characteristics of purebred Brazilian Holsteins which were reported to have an average coat thickness of 2.4 mm, with 12 mm, 65 mm, and 930 hairs cm^−2^ hair length, diameter, and density, respectively. Conversely, a coat layer with longer (25 mm) and denser hair (1,465 hairs cm^−2^) of lesser diameter (35 mm) was recorded in Holstein cows reared in temperate regions (49). The natural thermal conditions, such as levels of air temperature and solar radiation experienced by dairy cows in these regions, would certainly be the principal factors driving these differences. These reports emphasize how the genotype × environmental forces affected the dynamics of evolutionary changes on Holstein cattle population in tropical conditions.

Quantitative genetics, or the genetics of populations, is the study of characters which are not affected by the action of just a few major genes (49) and has diverse applications in animal breeding. The genetic correlation and hereditability (i.e., how faster a characteristic can be fixed in the next generations) of the physical properties of hair coat with productive and reproductive traits of the dairy cattle population can aid to derive the possibility for selecting better thermal tolerance traits together with improved productive traits. High values of coat thickness (>3 mm) were associated with a reduced conception rate in tropical Holstein cows due to their greater susceptibility to heat stress (50).

The genetic correlations between the hair coat traits and milk yield were tested in Brazilian Holstein cattle population (43, 44, 51) (Table 1). Their results suggest that majority of the hair coat traits are highly negatively correlated with milk yield. Furthermore, the lower heritability values for hair coat characteristics indicate that genetic changes as a result of individual selection will depend on the intensity of selection. On the other hand, high heritability (0.75) coefficient estimates for hair coat properties together with high negative genetic correlations of milk yield give an insight into a possibility of selection for increased milk yield together with better thermal resilience traits such as less dense coat with thick, short, and well-settled hairs.

**Table 1:** The genetic correlations between the hair coat traits and milk yield in Brazilian Holstein cattle population.

**Coat traits**	**C_**T**_**	**H_**L**_**	**H_**Den**_**	**H_**D**_**	**M_**yield**_**	**Heritability (h^**2**^)**
Coat thickness, C_T_	1.00	0.20	0.42	−0.06	−0.99	0.08
Hair length, H_L_	0.20	1.00	0.30	−0.65	−0.11	0.30
Hair density, H_Den_	0.42	0.30	1.00	0.10	−0.82	0.11
Hair diameter, H_D_	−0.06	−0.65	0.10	1.00	0.07	0.75
Milk yield, M_yield_	−0.99	−0.11	−0.82	0.07	1.00	0.35

*Data from 1,500 Brazilian Holstein cows (37, 38, 52)*.

Evaluating variables for genetic traits, especially of thermo-tolerance ability, requires intense data recorded in larger flocks which could improve its accuracy (49). The analyses using dense molecular markers greatly increased the information about the architecture of several complex traits. However, to overcome these limitations, crossbred animals are usually raised in tropical countries (53, 54).

On employing genomic tools, researchers were able to identify few specific genes associated to the heat tolerance of cattle. One of them is the SLICK haplotype (*Slick Hair* gene), which confers the animals with a short and sleek hair coat (12). Originally found in Senepol cattle, this gene was later identified in Carora cattle and introduced into Holstein by crossbreeding (55). Animals with the dominant allele have a very short and well-settled coat. Holstein cows with slick hair gene were reported to have better thermoregulatory ability than the non-slick animals and experienced less drastic depressions in milk yield during the summer (12). Such results were attributed to their better ability to evaporate sweat at the skin surface (56). Indeed Holstein cows kept in tropical regions with low coat thickness (<3 mm) and well-settled hairs have low diffusion resistance for water vapor and therefore greater capacity to evaporate sweat at the skin surface and dissipate heat to the surrounding environment (57).

In the context of evolutionary changes, employing statistical models and methods, it is possible to identify the changes pertaining to genotype and environment interaction within a given population of individuals possessing different phenotypes. The most widely used model in livestock breeding programs is the “animal model” or mixed model, which describes linear regression through a mixture of fixed and random effects. The term animal model is because the model is defined at the level of the individual animal (58), instead of the sire model (59). Basically, statistical analysis based on animal model estimates variance components and predicts additive genetic effects. Of the various statistical models, the most used model is the restricted livelihood approach (REML). The variance components can likewise be estimated using a Bayesian approach (60). The animal model and statistical method based on the REML were used to establish the genetic correlation between the hair coat traits and milk yield in Brazilian Holstein cattle population (43, 44, 51). The animal model is therefore the basis of studies on quantitative genetics for understanding the genetic and environmental effects on multiple traits.

The additive genetic variances due to production and heat tolerance were estimated using the model proposed by Ravagnolo and Misztal (61). This model adopted random regression on THI, containing repeatability of animal effects on test-day milk yields. The test-day model proposed by Ravagnolo and Misztal (61) compromised the effects of herd test date, milking frequency, DIM classes, age, general additive effect, random regression on THI for heat tolerance additive effect, general permanent environment, and the random regression on THI for a permanent environment. The variance components in this study were estimated using restricted maximum livelihood approach. It was observed that the heritability of milk yield in dairy cattle at THI below 72 was 0.17, and the additive variance for heat tolerance was zero. Furthermore, the additive variance of heat tolerance was as high as general effect for THI levels of 86, and the genetic correlation between the two effects was −0.36.

The broken line model was the traditionally used model to assess the productive response of animals to increasing heat load (62). This model was set with the assumption that production in animals remains constant within the thermo-neutral zone, with no variation in production with increasing temperature (within the thermo-neutral zone). However, once the THI exceeds the threshold limits, the production is predicted to decrease linearly (63). This model, however, may not reveal the true picture of the impact of heat stress on production due to the complex processes involved in animal production. Therefore, several authors have worked on improving the statistical models to assess the impact of heat stress in livestock, which could provide more flexible patterns (62, 64). A study was led by Carabano et al. (62) to explore the average and individual trends in response to milk production during heat stress in cattle reared under different climatic conditions and production systems in Europe. They assessed their objectives using different statistical models varying from the traditional broken line model to polynomial approximations.

Use of test-day models, which account for different recording schemes, utilizes incomplete lactation records and considers that the time-dependent effect for each test-day can be thought to be more efficient to evaluate thermo-tolerance in dairy cattle. Nguyen et al. (65) estimated the genomic estimated breeding value (GEBV) for heat tolerance for milk production traits like decline in milk, fat, and protein yield per unit increase of THI. They worked on implementing a plan to develop Australian genomic breeding value for heat tolerance (HT ABVg) for Holstein and Jersey dairy cattle. To estimate the HT ABVg, the effects of single-nucleotide polymorphism (SNP) on the selected heat tolerance traits were calculated following a series of steps. Firstly, the phenotypes for heat tolerance were calculated using the reaction norm model which estimated the rate of decline in milk, fat, and protein yield. In the model adopted, the data on milk, fat, or protein yield were set as fixed effects which also included several other factors like the herd test-day, year season of calving, parity, parity × an eighth-degree polynomial on DIM, a third-degree polynomial on age at calving, and stage of lactation × a linear polynomial on THI. A random regression on linear orthogonal polynomial of THI and a residual term were clubbed in the random effect. The THI threshold set for the model was 60, and the analysis was conducted using ASReml, which was done separately for Holstein and Jersey cattle. After calculating the pseudophenotypes for HT for sires and the SNP effects, the direct genomic values (DGV) for decline in milk, fat, and protein yields (kg) with heat stress (DGV_HT_trait_) and DGV in each trait (DGV_HT_ASI_) were derived. Finally, the HT ABVg was calculated using the following equation:

HTABGg = 100 + 5 × [DGV_HT_ASI_ – mean (DGV_HT_ASI_)] / SD (DGV_HT_ASI_)

Additionally, the reliability of HT ABVg in genotyped Holstein and Jersey bulls was estimated to be 38%, which was moderate and comparable with that of other economically important traits such as the feed saved. However, it is always recommended to further improve the reliability, which could be accomplished by increasing the size of the reference population. Furthermore, it would be ideal to identify and consider other heat tolerance traits like fertility and health (66), which would be a holistic approach to understand the heat tolerance ability of the animal considering its overall performance and net returns. Thus, HTABGg is considered a reliable model for the selection of superior animals for climate resilience.

In a recently concluded study, Hagiya et al. (67) assessed the genetic evaluation of heat tolerance in Holstein cows in Japan. The study was led using the herd-test-day model wherein the lactation curves on days in milk within age group were set as fixed effect while the general additive effect and heat tolerance of additive genetic effect were set as random effects. They used two models of Legendre polynomial function wherein the third-order functions were used for milk yield while the second-order functions were used for somatic cell score (SCS). Upon setting the threshold THI to 60, the mean heritability for THI = 78 was 0.20 and 0.28, which were lower than that of THI ≤60 that was 0.26 and 0.31. Additionally, the heritability estimates for SCS ranged from 0.08 to 0.10, which was similar to all heat stress conditions. Thus, from the study, it could be inferred that the EBVs of heat tolerance was changing in an undesirable direction.

In general, the use of statistical models to assess the genetic variables in a heat stress study varies depending on several factors starting from the data quality up to the possibility of incorporating computationally relevant traits. On the comparative basis of various statistical models discussed above, it was quite evident that the herd-test-day model provided a reliable approach to identify climate-resilient dairy cattle.

## Genomic Applications for Identifying Superior Dairy Cows Best

Conventional breeding strategies have played a potent role toward the production of superior animals; however, these methods were time consuming and most often would not consider all sources of genetic variability (68). The advanced molecular technologies mainly focused on the identification of molecular markers in DNA fragments, located in the genome, having the ability to assess the phenotypic variability (69). From the year 1980, several DNA markers were explored by geneticist to evaluate the genetic diversity of farm animals, with a vision to enhance livestock breeding programs (70). Advances in molecular biotechnology have led to the discovery of methodologies such as next-generation sequencing, microarray technology, whole transcriptome analysis, and genome-wide association studies aiding in efficient selection (3). Incorporation of such genetic information improved the accuracy of selection and hastened the genetic gain in the population (8).

Functional genomics is a field of molecular biology that describes the interaction and functions of genes and proteins using genome-wide approaches (70). Sheehy et al. (71) led an experiment on a functional genomics approach to investigate the functionality of six candidate genes located in a QTL interval for milk production traits on BTA6. They observed that, out of the four candidate genes exhibiting differential expression in bovine mammary tissue over the lactation cycle, only one gene *SPP1* (also known as osteopontin) played a significant role in modulating milk protein gene expression. Functional genomics could establish an assured link between gene expression and phenotype and is expected to have a major positive impact on sustainable livestock production. Incorporating such holistic approaches in heat-stressed dairy cattle could reveal novel pathways affected due to heat stress. Furthermore, it may also unfold the hidden pathways associated with adaptation during heat stress. There are several specific functional genomics approaches focusing on the DNA level (genomic and microarray technologies), RNA level (transcriptomic studies), protein level (proteomics), and metabolite level (metabolomics).

The microarray technology follows the principle of complementarity based on which target DNA sequence hybridized with the immobilized DNA molecules on the nylon chip or glass slide (3). The target sequences would be fragmented and labeled with fluorescent dyes which, on hybridization with the probe, would send signals which would be recorded by a detection system (72). Using this technique, a comprehensive profile could be generated for each animal based on the large number of polymorphisms, which would thereby aid in predicting the future performance and breeding value of an animal (73). Apart from studying the genotypic profile, the gene expression patterns under a particular situation or over a time period can be evaluated using cDNA microarray experiments (74). Thus, microarray technique could revolutionize selective breeding in animals. Collier et al. (74) adopted microarray analysis utilizing bovine-specific cDNA arrays to profile the gene expression in bovine mammary epithelial cell (BMEC) on exposure to acute heat stress. They assessed both the morphologic alterations and molecular dynamics in BMEC during heat stress.

Meuwissen et al. (75) proposed that the breeding value of an animal could be estimated from markers spanning its entire genome. Various SNP arrays were made available which, on implementation in dairy breeding, have successfully accelerated the rate of genetic gain in many milk production traits and have thereby changed the landscape of dairy animal selection (76). With the rising concern of impact of heat stress on dairy cattle production, it is of utmost importance to also consider adaptation traits while selecting animals.

The NGS technologies have been widely applied in dairy cattle to study the genetic variability underlying traits of economic interests (77). Gao et al. (77) conducted a study to assess the copy number variations (CNVs) having a potential association with milk composition traits in dairy cattle. They reported a total of 14,821 CNVs, some of which overlapped with 75 known QTLs and 235 functional genes having an association with milk protein and milk fat traits in dairy cattle. The NGS also plays a role in identifying candidate genes associated with climate resilience (3). This technology also gives an understanding of the molecular and cellular mechanisms associated with the thermal tolerance of ruminants in response to heat stress (3). Thus, this technology can play an important role in screening a population to identify genomic variants which could therefore be used in genomic selection.

Whole transcriptome analysis was used by many researchers to explore various fields of animal genetics (78). Liu et al. (79) explored the genes associated with heat tolerance and the molecular mechanisms adopted by Chinese Holstein dairy cows through transcriptomic analysis. They reported nearly 200 differentially expressed genes (DEGs) in heat-stressed cattle when compared to their control. They also observed several hub genes like OAS2, MX2, IFIT5, and TGFB2 to be significantly enriched in immune effector process.

Li et al. (80) studied the expression of long non-coding RNA (lncRNA) using the deep RNA sequencing approach of NGS platform in heat-stressed and non-heat-stressed Chinese Holstein cattle. Their analysis provided a comprehensive report of lncRNAs, covering their expression and also analyzing its biological functions. Such studies thereby open up newer pathways to elaborately assess the genetic potential of an animal to produce optimally amidst the stressful environmental scenario. Table 2 describes the different biotechnological tools to identify various genes associated with climate resilience in dairy cows.

**Table 2:** Biotechnological and statistical modeling methodologies to identify different genes associated with climate resilience capacity in dairy cattle.

**Biotechnological/statistical modeling methodologies**	**Breed**	**Targeted gene**	**References**
qRT-PCR	Holstein Friesian	*SPP1*	(44)
RNA-Seq	Holstein	*IGF1R, ADCY4, CISH, DDIT3, STAT1, FURIN, IGFBP6, INSIG1, INSIG2, NMI, PAPPA, SCAP, SOCS5, SREBF1, SRPR, STAT3, STAT5A, STAT5B*, and *STAT6*	(81)
RNA-Seq	Holstein-Friesian	*CYP11A1*	(55)
GWAS	Canadian Holstein	*DGAT1, CPSF1*	(7)
GWAS	Crossbred cows	*GNA14* and *LRRC4C*	(64)
GWAS	Italian Holstein	*HSF1, MCAT*	(65)
NGS	Holstein	*INS, IGF2, FOXO3, TH, SCD5, ALNT18, GALNT16, ART3, SNCA WNT7A*	(51)
PCR amplicon sequencing	Native Turkish cattle (Yerli Kara, Boz irk, Yerli Güney Sarisi, Güney Dogu Anadolu Kirmizisi and Dogu Anadolu Kirmizisi) and Holstein	HSP70.1	(82)
RNA-Seq	Holstein	*HSPA1A, HSPH1, HSPA8, DNAJA1*, and *CDK1*	(83)
Qpcr	Tharparkar	*IL2, IL6, TLR2, TLR4*	(84)
GWAS	Butana and Kenana	*HSF5*	(80)
WGS	Holstein, Hanwoo, and N'Dama	*5S_rRNA, 7SK, ARFGAP3, SNORA70, U1, U6*, and *U6atac*	(79)
GWAS	Holstein	U1, *NCAD*, SNORA19, *RFWD2, SCARNA3, SLCO1C1, PDE3A, KBTBD2, LSM5*, and *GOT1*	(66)

## Scopes of Genetic Models for Identifying Climate-Resilient Dairy Cows

### Genome-Wide Association Study Applications in the Dairy Sector

In livestock, genome-wide association studies (GWAS) have been used extensively to map the QTL of important traits like production traits (85, 86), reproduction traits (87), adaptive traits for thermo-tolerance (88), methane emission traits (89), and so on.

Iso-Touru et al. (90) conducted the GWAS for milk production traits in Nordic Red cattle by imputing whole-genome sequence variants which, despite being a robust procedure, was found to be cost-effective in expanding the information available and also widening the knowledge of causative mutations that influence the milk yield traits in cattle. In their study, they could identify 3,594, 755, and 85 SNPs having a significant association with fat yield, milk yield, and protein yield, respectively. Such studies open up endless possibilities to study the quantitative trait architecture more closely for their effective use in cattle breeding program.

Yodklaew et al. (91) conducted a GWAS to assess the milk yield per lactation, persistency, initial milk yield, and age at first calving in a multibreed dairy cattle population in Thailand. The study was conducted using a set of SNP markers that would be in common between GeneSeek Genomic Profiler low-density bead chips and GeneSeek Genomic Profiler high-density bead chips. A total of 8,096 SNPs, common between the two chips, were utilized by the single SNP association analyses. Of these, a total of 366 markers were found to have a significant association with the traits studied, which could thereby aid in the genetic selection of superior animal for future breeding.

Another study was conducted in Holstein dairy cattle to establish the genetic correlation and GWAS of the length of 305-days milk yield for the first lactation (FM305), length of productive life, and days open (92). In total, 23 QTL regions, spread across chromosomes 1, 4, 5, 8, 15, 26, and X, were found to be positively associated with all the three traits. Based on these results, the authors concluded that these QTLs may be considered for marker-assisted selection to improve the productive performance of Holstein dairy cattle. Until the recent past, most of the genomic studies primarily focused on assessing production traits in dairy animals due to its economic significance. However, with the projected impact of climate change on livestock production, especially the dairy sector, research efforts are currently in progress to explore the application of these technologies to evolve a breeding strategy using marker-assisted selection involving both productive as well as adaptive traits. Such efforts may help in the identification of candidate genes for heat stress in dairy cattle in the near future.

Dikmen et al. (93) performed a GWAS for rectal temperature of lactating Holstein cows during heat stress with an objective to identify potential SNPs that would serve as a QTL for the same trait. All the relevant data and variables were recorded in 4,447 cows, and the genotypes in 1,440 cattle (107 cows and 1,344 bulls) were obtained through Illumina BovineSNP50 BeadChip with 39,759 SNP. They reported QTL markers across five chromosomes (BTA 24, BTA 16, BTA 5, BTA 4, and BTA 26) which either included or was nearby certain functional regions or genes such as U1 spliceosomal RNA, *cadherin-2* (*NCAD*) gene, small nucleolar RNA (SNORA19), ubiquitin-protein ligase (*RFWD2*), Cajal body-specific RNA 3 (*SCARNA3*), solute carrier organic anion transporter family member 1C1 (*SLCO1C1*), phosphodiesterase (*PDE3A*), kelch repeat and BTB domain-containing protein (*KBTBD2*), U6 snRNA-associated Sm-like protein LSM5 (*LSM5*), and glutamine-oxaloacetic transaminase, soluble (*GOT1*). These genes were predicted to have a direct or indirect role in regulating thermal stress. Thus, from this study, it was concluded that these SNPs could also be useful in the genomic selection of climate-resilient dairy animals.

## Genomic Breeding Model for Dairy Cattle Selection

The extent of decline in milk production due to heat stress varies between animals which have a low to moderate level (0.13–0.23) of heritability (94). This trait, reduction in milk yield due to heat stress, could be a potential indicator for heat stress. Although traditional selection programs involving phenotype and pedigree information did brought in substantial improvement in milk yield, still this approach had lower genetic gain due to the long generation interval and lower heritability for the resilience traits in dairy cattle. This opened up the gateway for more refinements in breeding strategies, and genomic selection could therefore be an ideal alternative to overcome this limitation to select elite animals based on their GEBV for heat tolerance (65). This methodology uses genome-wide DNA markers which capture the effects of several genomic variations that influence complex traits.

Nguyen et al. (65) adopted the genomic best linear unbiased prediction (GBLUP) to calculate GEBV for heat tolerance in Holstein and Jersey cattle for milk production traits (milk, fat, and protein yield). Their study revealed improved accuracy for breeding values using genomic selection. As per their study, the rate of decline in production during heat stress was used as a variable to define tolerance to heat stress. On incorporating a random regression model with a common THI threshold of 60 for all cows, heat tolerance was estimated. Additionally, the daughter trait deviations of their sires were defined using the slope solutions for cows. The accuracy of GBLUP for genomic prediction for Holstein and Jersey based on parity and number of sires and cows are 0.39–0.57 and 0.44–0.61, respectively. Furthermore, studies conducted by Garner et al. (83) also revealed that cattle predicted to be heat tolerant by genomic breeding values (0.48) had fewer declines in their milk production in addition to reduced increment of body core temperature on exposure to a simulated heat wave event in comparison to predicted heat-susceptible cows. Therefore, this approach could hasten the development of climate-resilient dairy cattle.

GBLUP has been proven to be more accurate to predict the breeding value of an animal and further improve selection accuracy and gain. However, this methodology assumes genomic markers to contribute equally toward the variability of a trait (75), which may not necessarily be true always. When only broader effects are included in the model, the accuracy of predicting genetic values was only 0.32. However, on the basis of the best linear unbiased prediction of haplotype effects which assumed equal variances to each 1-cM chromosomal segment, there was a yield of accuracy of 0.73, while Bayesian methods that assumed a prior distribution of the variance associated with each chromosome segment increased this accuracy to 0.85 (75). Most of the genetic markers associated with complex traits could be clustered in genes which are involved in interconnected biological pathways and networks (82).

Several other statistical models have been used in GWAS like principal component regression (95), partial least squares regression (96), LARS (97), LASSO (84), BLUP including a genomic relationship matrix (98), fixed regression using least squares, random regression BLUP, Bayesian regression, partial least squares regression, support vector regression, and so on (99). However, most of these approaches were adopted to estimate the genetic variables associated with production traits, while their incorporation in evaluating thermo-tolerance in dairy cattle was limited. Nevertheless, researchers are now venturing into the field of climate change and livestock production wherein attempts have been made to study the phenotypic plasticity of dairy cattle and predict the genetic components associated with thermo-tolerance.

Mateescu et al. (100) conducted a study to estimate the body temperature plasticity in six breed groups of multibreed Angus-Brahman heifers (ranging from 100% Angus to 100% heifers) on exposure to heat stress. So as to achieve their objective, the authors adopted two main statistical approaches. Firstly, the reaction norm variables for each breed on exposure to a specified heat stress condition were estimated using a random regression model. Subsequently, the response of the animals in each group to different environmental heat loads were evaluated using repeated-measures mixed model. Sigdel et al. (101) led a study with a primary objective of estimating the genetic components of milk yield traits in Holstein cows under heat stress using multi-trait repeatability test-day models with random regression as a function of THI. In their model, the herd-test-day and DIM classes were categorized as fixed effects, while general and thermo-tolerance additive genetic effects and permanent environment were taken as random effects. These models were effective in estimating the genetic components of heat stress effects which also revealed a negative correlation between general and thermo-tolerance additive effects (−0.18 to −0.68), highlighting the higher susceptibility of high producers to heat stress. The second objective of their study was to identify genes and pathways involved in milk production during heat stress in cattle using genome-wide scans. Using single-step BLUP model (ssGBLUP), they assessed the whole-genome association in Holstein cows. The ssGBLUP model used replaced the inverse of the pedigree relationship matrix (A−1) (used in classical BLUP model) with the inverse of the realized relationship matrix (H−1). Therefore, incorporating the H-1 combined both pedigree and genomic information, thereby substantiating the results. Through this study, candidate genes and associated pathways were also identified. Therefore, updating the biological knowledge of functional genomic regions for production and adaptation traits could enable the effective usage of this methodology to develop climate-resilient dairy cattle.

### Selection Signature Applications in the Dairy Sector

According to the theories in population genetics, alleles that are favorably selected are either lost or increase in frequency in the population until it gets fixed (102). Selection signatures help to demarcate such functionally important regions in the genome using various statistical approaches (103). A number of methods have been developed, like the extended haplotype homozygosity and relative extended haplotype homozygosity (88), to detect recent selection: Tajima's D (104) and Fay and Wu's H-test (105) to identify selected mutations, F_ST_ and pairwise F_ST_ (106, 107), integrated Haplotype Score (iHS) (107) which measures large-scale allele frequency differences among populations, Rsb test (108) which identifies the loci under selection, and hapFLK (109) which also accounts for the hierarchical structure of the sampled populations. These approaches have been used in livestock to detect potential loci and candidate genes that have undergone positive selection and influenced complex traits (102).

Schwarzenbacher et al. (102) combined iHS and a locus-specific permutation-based iHS with whole-genome association study to identify regions which influence complex traits in dairy cattle. Through their study, they could identify 1,600 SNPs from a total of 34,851 SNPs, exhibiting selection signatures. The results obtained from this study were reliable, with lower false positive values, and hence increased the efficacy to detect potential loci that have an influence on production traits in dairy cattle. Taye et al. (110) conducted a study to explore genomic regions/genes under selection using population statistics methods like Tajima's D, XP-CLR, and XP-EHH in Holstein, Hanwoo, and N'Dama cattle. A total of 441, 512, and 461 genes were found to be under positive selection in Holstein, Hanwoo, and N'Dama cattle, respectively. These genes were found to have influence on various economic traits like milk production, reproduction, and meat and carcass traits. Adopting similar models to identify potential selection signatures in heat-stressed dairy cattle would complement to build the database for developing climate-resilient dairy cattle.

A study was led by Bahbahani et al. (109) to identify signatures of positive selection in two African dairy cattle breeds. They genotyped Butana and Kenana breed of zebu-type dairy cattle using the BovineHD Genotyping BeadChip and explored for positive selection signatures using iHS and Rsb analysis. The statistical analysis revealed 87 and 61 selection regions in Butana and Kennaee breeds, respectively, which included a number of genes and QTL having an association with different traits like milk production, immunity, thermotolerance, and reproduction. They identified a total of 30 candidate regions under positive selection which overlap with regions identified in commercial dairy cattle by other researchers (Holstein Friesian, 100; Jersey, 98). Heat shock transcription factor family member 5 (*HSF5*), which plays a role in adaptation to heat stress, was also identified in a candidate region in Butana cattle. Such studies aid in identifying potential genes and QTLs having a significant association with various production and functional traits in cattle which are under positive selection. Such information plays an influential role while deciding breeding programs in dairy cattle to improve their production.

Local cattle breeds, though not highly productive, are well-acclimatized to the local environment. Yurchenko et al. (14) conducted a study on nine native cattle breeds in Russia, which are adapted to harsh climate, with the primary objective to identify candidate regions under positive selection by performing a genome scan using haplotype-based statistic (hapFLK) (111). Additionally, they followed composite measures of selection, for better results, using a combination of five genome-wide statistics: F_ST_, haplotype homozygosity (H_1_), modified haplotype homozygosity (H_12_), Tajima's D index, and nucleotide diversity (Pi). On comparing the data with that of other breeds of both European and Asian origins, they identified some novel and previously reported candidate genes having a significant association with economically important traits and environmental adaptations in cattle. They identified some putative selection signature regions covering some important genes influencing milk production traits in cattle like *DGAT1* (influencing milk fat content), *ABCG2, LCORL, GHR*, and *NCAPG* (affecting milk yield), *FKBP2* (associated with milk protein percentage and yield), *KLHL1* (influencing milk yield and lactation persistency), *KDM5A* (associated with fatty acid level), and many more. Furthermore, their study highlights the usage of such candidate regions while improving crossbreeding programs in an effort to improve the commercial breeds such that they could acclimatize better to the harsh environment and thus perform better.

Using suitable selection signature methods, one can identify selection signatures, thereby detecting candidate genes and their location, which have a potential role in various economical traits in cattle. This would further give a better insight into the history of selection and also the genetic adaptability of the animal/population to environmental distress (112).

## Biomarkers For Identifying Thermo-Tolerant Dairy Cow

Genetically selecting thermal-resilient animals would help to improve the production of dairy cattle during the hot season, and the primary mandate for this is to understand the genes and QTL regions having a role in heat stress regulation (112). There are few reports which identified such biomarkers in the bovine genome as having association with thermo-tolerance [*HSP70.1* (91); *ATP1B2* (92); *HSF1* and *HSPA6* (90); *HSP90AA1* (94); *TNF1/4, IL2/6* (83); *EIF2AK4* (40); *KIF9, SELENBP1, XDH* (45)].

*HSP90AA1* belongs to the group of heat shock proteins (HSPs) which play a vital role in protecting cells from environmental stress. Kumar et al. (113) conducted a study in Sahiwal cows to identify SNPs in exons 3, 7, 8, and 11 of *HSP90AA1* and study its association with thermal tolerance. They reported a total of five SNPs (A1209G, A3292C, T4935C, T5218C, and A5224C) in this breed of indigenous cattle, among which two, A1209G and A3292C, had a significant association with heat tolerance traits. Bharati et al. (114) likewise studied the expression dynamics of *HSP70* during chronic heat stress exposure in another indigenous Tharparkar breed. On acclimatizing six cattle at a thermoneutral zone for an initial period of 15 days, they were exposed to an environmental temperature of 42°C for the next 23 days, which was finally followed by a recovery period of 12 days. During the course of the study, PBMCs were collected at regular intervals from the animals, from which mRNA was isolated for expression study. It was observed that the *HSP70* mRNA expression was significantly increased and had two peaks on day 17 and day 32 (2nd and 17th days of the thermal challenge, respectively). It was inferred that the first peak would be to ameliorate the negative effects of thermal stress and the second would be to tackle the chronic exposure to heat stress, and thereby this gene could be used as a biomarker to select thermo-tolerant cattle (114). Table 3 describes the different biomarkers for assessing heat stress resilience in dairy cattle.

**Table 3:** Different biomarkers for climate resilience in dairy cattle.

**Genes/variants/biomarkers associated with climate resilience**	**Source**	**Positions in the genome**	**Gene ID**	**Biological function**	**Pathways**
HSP70.1	Blood	Chromosome 23	282254	Protect cells from thermal damage and apoptosis	Apoptosis Modulation and Signaling and HIF1Alpha Pathway
ATP1B2	Blood	Chromosome 19	282562	Role in reducing the rectal temperature	Cellular response to heat stress
HSF-1	Blood	Chromosome 14	506235	Transcriptional activation of the heat shock response	Cellular response to heat stress
HSPA6	Blood	Chromosome 3	539835	Role in cellular thermo-tolerance	Cellular response to heat stress
IL2	Blood	Chromosome 23	280822	Regulation of the immune response	Cell mediated immune response pathway
IL6	Blood	Chromosome 4	280826	Regulation of the immune response	Cell mediated immune response pathway
TLR2	Blood	Chromosome 17	281534	Regulation of the immune response	TLR signaling pathway
TLR4	Blood	Chromosome 8	281536	Regulation of the immune response	TLR signaling pathway
HSPA1A	Blood	Chromosome: 23	282254	Protect cells from thermal damage and apoptosis	Cellular response to heat stress
HSPH1	Blood	Chromosome: 12	507165	Protect cells from thermal damage and apoptosis	Cellular response to heat stress
HSPA8	Blood	Chromosome: 15	281831	Protect cells from thermal damage and apoptosis	Cellular response to heat stress
DNAJA1	Blood	Chromosome: 15	528862	Thermal Adaptation	Cellular response to heat stress
GH1	Blood	Chromosome: 28	280804	Growth regulation	Hormone-response pathways involved in energy metabolism
FASN	Blood	Chromosome: 19	281152	Role in lipogenesis	Lipid metabolism pathway
HSP60	Blood	Chromosome: 2	511913	Protect cells from thermal damage and apoptosis	Cellular response to heat stress
TLR3	Blood	Chromosome: 27	281535	Pathogen recognition and activation of innate immunity	TLR signaling pathway
HSF5	Blood	Chromosome: 19	784069	Lactation persistency	Enrichment pathways for lactation persistency
AQP5	Blood	Chromosome: 5	782368	Role in cold acclimation	Cellular response to cold stress
RAD50	Blood	Chromosome: 7	788127	Candidate gene for harsh environmental adaptation in cattle	Immune system pathway
RETREG1	Blood	Chromosome: 20	540068	Role in adaptation to harsh environment	Molecular response to heat stress
HSP90AA1	Blood	Chromosome: 21	281832	Heat tolerance in dairy cattle	Cellular response to heat stress
PRLR	Hair Follicle	Chromosome: 23	280901	Heat tolerance of temperate dairy cattle	Prolactin pathway in regulating heat tolerance
PPARG	Hair Follicle	Chromosome: 22	281993	Regulating lipid and glucose metabolism	Lipid metabolism pathway in heat stressed animals
EIF2AK4	Ear Tissue	Chromosome: 10	513829	Role in thermo-tolerance	Cellular response to heat stress
SELENBP1	Mammary Epithelial Cells	Chromosome: 3	510154	Role in milk fat depression	*Selenium metabolism and selenoproteins pathway*
XDH	Mammary Epithelial Cells	Chromosome: 11	280960	Role in milk synthesis	Milk synthesis pathways
SPP1	Mammary Epithelial Cells	Chromosome 6	281499	Improving lactation persistency	Enrichment pathways for lactation persistency

A transcriptome analysis in Holstein calves was carried out by Srikanth et al. (112) using RNA-seq to characterize genes and pathways that respond to heat stress. The calves were subjected to varied ranges of temperature and humidity, and their skin and rectal temperature values were recorded. On RNA-seq analysis, 8,567 DEGs were identified for heat stress, among which 465 were up-regulated and 49 were down-regulated. On further analysis, they reported *HSPA1A, HSPH1, HSPA8, DNAJA1*, and *CDK1* to be the top five up-regulated genes. They also reported the differential expression of 31 transcription factors, 13 transcriptional cofactors, and six chromatin remodeling factors. Based on the results of this study, candidate genes may be selected for future breeding programs.

A GWAS for heat stress response in Gir × Holstein crossbred cattle was conducted by Otto et al. (115). The study was led with an aim to detect SNPs influencing heat stress response and also to identify the candidate genes for thermo-tolerance. For the study, rectal temperature (RT) was recorded in heat-stressed animals, and the difference between two consecutive RT (ΔRT) was used as the dependent variable. A total of six SNPs were found to be significantly associated with ΔRT which were distributed across three QTL regions on BTA17. Further analysis revealed *LIF, OSM, TXNRD2*, and *DGCR8* as candidate genes influencing the biological processes which alleviate the harsh effects of heat stress.

Yodklaew et al. (91) conducted a GWAS for milk characteristics in a Thai multibreed population and had identified 366 markers having a significant association with these traits. These markers, however, may not be observed in another population as the environmental temperature may affect its expression. Hence, it is always necessary to consider the climatic conditions in a particular region before incorporating their breeding strategies (116, 117).

Knowledge and access to genomic information on an individual has been expanding exponentially. Garner et al. (83) genotyped 390 Holstein Friesian cows and estimated their GEBV from which top 24 heat-tolerant (HT) and top 24 heat-susceptible (HS) heifers were selected for validation. On subjecting these animals to controlled climate chamber study, they observed that the decline in daily milk yield from the baseline period until day 4 of heat stress was significantly lesser (*P* = 0.023) in HT cows (12.5% decline from baseline) than the HS cows (17.4% decline from baseline). Incorporation of such holistic approaches of GWAS which would depict candidate genes followed by its incorporation into GEBV will aid in the selection of climate-resilient cows having the ability to maintain its production. This would further reduce the cost on expensive management practices and reduce production losses due to rising temperature (118). Thus, these efforts, apart from ensuring the livelihood security of the farmers, may also help to relieve the stress to the animals and improve their well-being.

### Crossbreeding as a Potential Breeding Strategy for Establishing Climate Resilience in Dairy Cattle

The human intervention to improve the resistance of *Bos taurus* cattle to environmental stress factors dates back 4.2 thousand years. Genome-wide analysis of 67 ancient Near Eastern cattle, *B. taurus*, revealed that a later region-wide Bronze Age shift indicates rapid and widespread introgression of Zebu, *Bos indicus*, from the Indus Valley. This was not attributed to the simple diffusion between these two populations (119). It was suggested that this process was rather adopted by cattle herders aiming to improve the resilience of *B. taurus* cattle population to high-intensity and frequent droughts at that time. The hybrid *B. taurus*–*B. indicus*, therefore, may have enabled the survival of communities and perhaps facilitated the expansion of herding into more peripheral regions.

Over the centuries, thermal environmental stressors have compromised the productivity and welfare of high-producing *B. taurus* dairy cattle living in tropical climates or/and even those in temperate regions that frequently encounter extreme weather events (35). In the tropical climate of Brazil, for example, with the development of the dairy industry in the early twentieth century (1920s and 1930s), there were attempts to introduce several European cattle breeds such as Holstein, Jersey, and Brown Swiss. Although more productive, these animals were not able to adapt to the new environment by not expressing their genetic potential due to high susceptibility to heat stress and diseases, which was exacerbated by the extensive farming system. Under such conditions, the animals were faced with high levels of solar radiation and mean radiant temperature in addition to tick infestations and other diseases (120).

Around the 1940s, Brazilian farmers began to cross the Gyr cattle (an imported *B. indicus* breed) with the Holstein (121), aiming to produce a phenotype (F1) with better thermal tolerance and yet good productive performance. Analyses of the milk yield of Holstein × Gyr crosses an average of 2,574 kg per 305 days, which was significantly higher than the 1,600 kg yielded by a natural population of Gyr in India (121). As a result, most of the dairy cattle population in Brazil is now represented by crossbreds (mainly 5/8 Holstein × Gyr). By this approach, it was established that such crossbreds possessed both productive as well as adaptive characteristics as compared to the contemporary Holstein cattle of temperate regions (122).

Heat tolerance is determined by the relationship between metabolic heat production and the ability to dissipate body heat by employing evaporative cooling mechanisms, mainly through sweating (35). There are clear evidence of crossbreeding improving heat tolerance of dairy cattle living in hot conditions; for example, the mean coat thickness (~2.7 mm) was similar in Gyr, Gyr × Holstein F1, >75% Holstein × Gy, and tropical purebred Holstein cattle population (42, 123). In fact, researchers propose crossbreeding to be a potential strategy to impart both productive and adaptive potential to indigenous and pure breeds, respectively (47, 48).

## Proposed New Breeding Strategy for the Dairy Sector

In the current climate change scenario, researchers are now focusing to incorporate thermo-tolerance and low methane emission traits into breeding programs which were primarily looking into productive traits. Such a holistic breeding approach would ensure sustainable livestock production. Limited studies have been conducted in this aspect. However, there are a few reports of *h*^2^ estimates for low methane emission and thermo-tolerance in cattle. Dikmen et al. (93) estimated the *h*^2^ of rectal temperature (using BLUP) and its genetic correlations with production and reproduction traits (with Gibbs sampling) during the summer in 1,695 lactating Holstein cows that were sired by 509 bulls. As per their report, rectal temperature was found to have a moderate *h*^2^ of 0.17 ± 0.13, having genetic correlation with milk production traits. Lassen and Løvendahl (124) likewise studied the *h*^2^ of three CH_4_ phenotypes: the CH_4_ and CO_2_ ratio in the breath of the cows (CH_4__RATIO), estimated quantified amount of CH_4_ (in g/d) recorded over a week (CH_4__GRAMSw), and CH_4_ intensity, which was grams of CH_4_ per liter of milk produced (CH_4__MILK). The *h*^2^ of CH_4__GRAMSw and CH4_MILK was estimated to be 0.21 ± 0.06, while that of CH^4^_RATIO was 0.16 ± 0.04. They also observed that methane production has a strong negative correlation with milk production traits.

## Concluding Remarks

This review has projected the information pertaining to the impacts of heat stress on milk production of dairy cows and the various genomic tools that are available to identify climate-resilient dairy cows. The different functional and economical traits which govern milk production can be identified using advanced biotechnological tools and statistical models to improve thermo-tolerance in dairy cattle. Although several refinements have taken place in these areas over the years, the GWAS and selection signatures were the most preferred options for researchers across the globe to identify robust animals to withstand adversities associated with climate change. These tools can be used to detect potential loci and candidate genes that have undergone positive selection in complex milk production traits of dairy cattle during exposure to tropical climate. Thus, few biomarkers such as *HSP70.1, ATP1B2, HSF1, HSPA6, HSP90AA1, TNF1/4, IL2/6, EIF2AK4, KIF9, SELENBP1*, and *XDH* were found to be associated with thermo-tolerance in dairy cows. Furthermore, GWAS analysis revealed *LIF, OSM, TXNRD2*, and *DGCR8* as candidate genes influencing the biological processes which alleviate the harsh effects of heat stress in heat-stressed animals. Incorporation of such candidate genes through marker-assisted selection program can improve the climate resilience capacity of dairy cattle.

## Future Perspectives

Although tremendous progress has been made in the last decade by employing these advanced molecular biology tools to identify various QTLs and genes associated with thermo-tolerance in dairy cattle, such research is still in infancy state. Most of these studies stopped at identification of various biomarkers in the laboratory condition, and it is still a long way to go before these technologies are implemented to develop robust animals which have the ability to survive in adverse environmental conditions without compromising their production potential. For this to happen, genomic selection breeding has to be intensified based on the already identified biomarkers/traits governing heat stress regulation and uncompromised milk production in dairy cattle. These advanced biotechnological tools should also be used to identify more traits pertaining to adaptation, production, and low methane emission. Based on this knowledge, efforts are also equally needed to develop a robust animal which has the ability to survive in multiple locations, produce milk optimally, and emit less methane per unit of feed consumed. Such efforts not only can ensure the livelihood security of poor and marginal farmers but also can make the livestock enterprises more profitable. As the world is battling to meet the huge food demand of the growing human population by 2050, such efforts of developing multi-location thermo-tolerant dairy cattle breeds may prove highly beneficial to ensure food security.

## Author Contributions

MS and VS conceptualized the review and developed the outline. MS, VS, PM, MN, VF, and AM prepared the manuscript covering various sections. SK and RB edited and corrected the manuscript. All authors proof read and approved the manuscript.

## Conflict of Interest

The authors declare that the research was conducted in the absence of any commercial or financial relationships that could be construed as a potential conflict of interest.
